# Graves disease complicated by agranulocytosis and thrombocytopenia in a 12-year-old girl

**DOI:** 10.1210/jcemcr/luag271

**Published:** 2026-09-22

**Authors:** Yutaro Yoshiki, Yuki Yamano, Takatoshi Maeyama, Tamaki Wada, Yuri Etani, Masanobu Kawai

**Affiliations:** Department of Gastroenterology, Nutrition and Endocrinology, Osaka Women's and Children's Hospital, Izumi 594-1101, Japan; Department of Gastroenterology, Nutrition and Endocrinology, Osaka Women's and Children's Hospital, Izumi 594-1101, Japan; Department of Molecular Genetics and Endocrinology, Research Institute, Osaka Women's and Children's Hospital, Izumi 594-1101, Japan; Department of Gastroenterology, Nutrition and Endocrinology, Osaka Women's and Children's Hospital, Izumi 594-1101, Japan; Department of Gastroenterology, Nutrition and Endocrinology, Osaka Women's and Children's Hospital, Izumi 594-1101, Japan; Department of Gastroenterology, Nutrition and Endocrinology, Osaka Women's and Children's Hospital, Izumi 594-1101, Japan; Department of Molecular Genetics and Endocrinology, Research Institute, Osaka Women's and Children's Hospital, Izumi 594-1101, Japan

**Keywords:** Graves disease, child, agranulocytosis, thrombocytopenia

## Abstract

Graves disease is the most common cause of hyperthyroidism in children. Although neutropenia and thrombocytopenia have been reported in adults with untreated Graves disease, these complications are rare in children. Here, we report a pediatric case of Graves disease complicated by agranulocytosis and thrombocytopenia at diagnosis. A 12-year-old girl presented with increased appetite, irritability, fever, and petechiae. Laboratory examination revealed thyrotoxicosis, and this was associated with agranulocytosis and thrombocytopenia. There was no history of antithyroid drug use. The diagnosis of Graves disease was established based on positive thyroid-stimulating hormone receptor antibodies and thyroid scintigraphy findings. After initiation of a β-blocker and potassium iodide, neutrophil and platelet counts gradually improved in association with the resolution of thyrotoxicosis, which allowed the subsequent introduction of methimazole. The β-blocker and potassium iodide were sequentially discontinued. Blood cell counts remained within the normal range thereafter, and thyroid function has remained well controlled with methimazole monotherapy. This case suggests that thyrotoxicosis itself is associated with cytopenia and provides valuable insight for clinicians when selecting a therapeutic strategy for Graves disease complicated by cytopenia.

## Introduction

Graves disease (GD) is the most common cause of hyperthyroidism in children and adolescents [1]. Although GD typically presents with weight loss, tachycardia, and goiter [1], cytopenia at presentation has also been described, likely related to thyroid hormone excess and/or associated autoimmune mechanisms [2, 3]. In adults, neutropenia has been reported before the initiation of treatment in 14.1% of individuals with newly diagnosed GD, and neutrophil counts generally improve after treatment as euthyroidism is achieved [4]. Thrombocytopenia (platelet count <15.0 × 10^4^/µL [International System of Units (SI): <150 × 10^9^/L]) has also been reported in untreated hyperthyroidism, with platelet counts improving with treatment [5, 6]. In children, pretreatment neutropenia has also been reported [7], but severe cytopenias (agranulocytosis) and thrombocytopenia appear to be rare, with only limited pediatric reports [2, 8, 9]. Here, we report a pediatric case of GD complicated by agranulocytosis and thrombocytopenia at diagnosis with a review of the relevant literature.

## Case presentation

A previously healthy 12-year-old girl presented with a 2-week history of increased appetite and irritability. Three days before referral, she developed fever and generalized fatigue and noted neck swelling. One day before referral, petechiae appeared, and blood tests at the referring clinic suggested hyperthyroidism. Her family history was unremarkable. On referral, her height and weight were 151.9 cm (−0.2 SD) and 35.5 kg (−1.4 SD), respectively. She was alert, with a temperature of 37.5 °C and a heart rate of 130 beats/minute. She had no exophthalmos, but a diffuse goiter was present. Palmar hyperhidrosis was noted, and petechiae were observed in the oral cavity and on the skin from the neck to the abdomen.

## Diagnostic assessment

**Figure 1:**
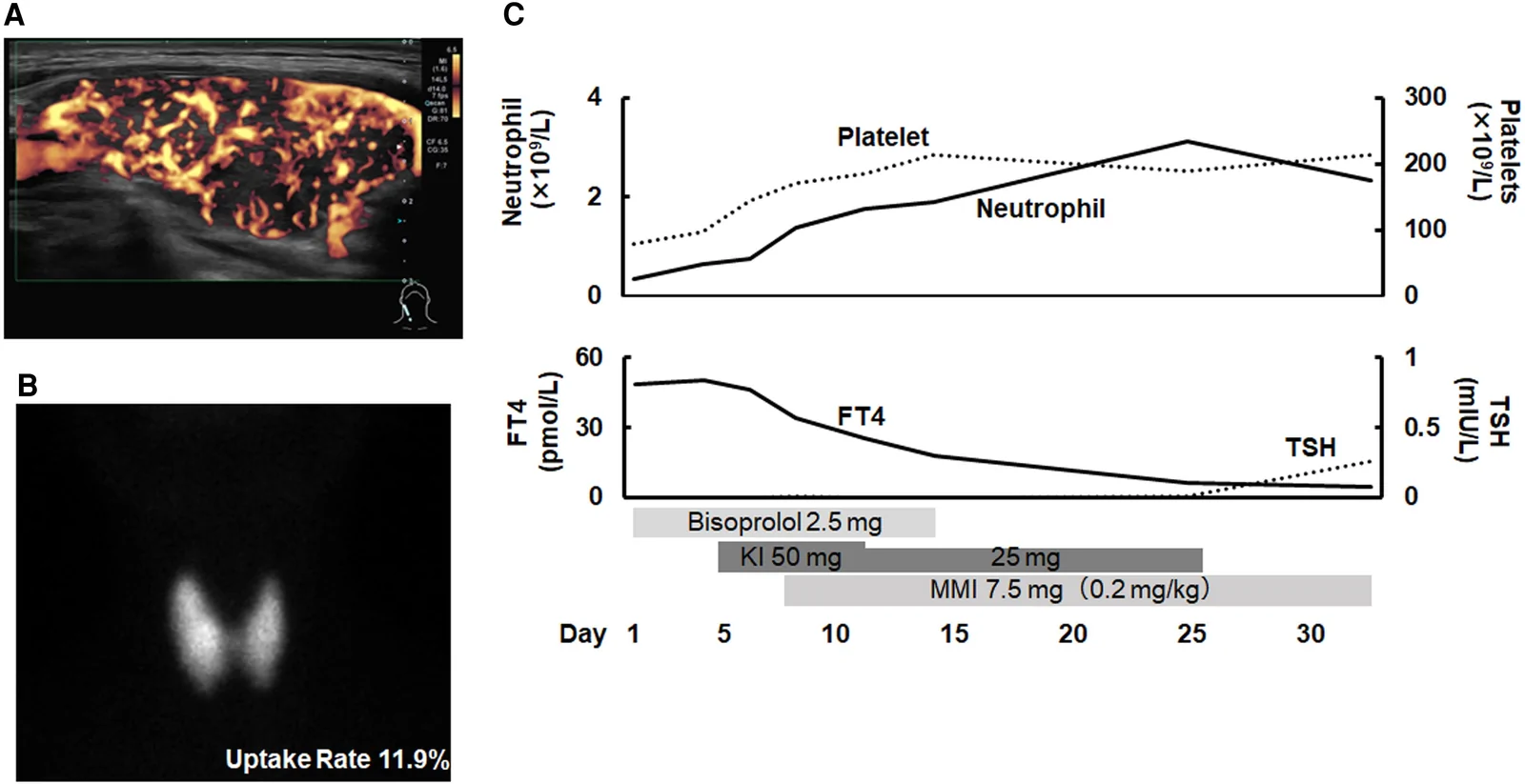
Imaging findings and clinical course of the patient. (A) Color Doppler ultrasonography demonstrating increased intrathyroidal blood flow. (B) Technetium-99m thyroid scintigraphy demonstrating increased thyroid uptake (11.9%). (C) Clinical course after admission changes in thyroid hormone levels and blood cell counts during hospitalization are shown. Treatment with a β-blocker was initiated on admission, followed by KI therapy after the diagnosis of Graves disease. Improvement in thyroid function was accompanied by gradual recovery of neutrophil and platelet counts, allowing for the subsequent initiation of MMI.

Laboratory findings are summarized in Table 1. The white blood cell count was 1.7 × 10^3^/µL (SI: 1.7 × 10^9^/L) (reference range, 4.5-13.0 × 10^3^/µL [SI: 4.5-13.0 × 10^9^/L]) with an absolute neutrophil count of 0.36 × 10^3^/µL (SI: 0.36 × 10^9^/L) (reference range, 1.5-8.0 × 10^3^/µL [SI: 1.5-8.0 × 10^9^/L]), and the platelet count was 8.0 × 10^4^/µL (SI: 80.0 × 10^9^/L) (reference range, 13.0-40.0 × 10^4^/µL [SI: 130-400 × 10^9^/L]), indicating bicytopenia with agranulocytosis. Serum free triiodothyronine (FT3) and free thyroxine (FT4) levels were elevated at 14.22 pg/mL (SI: 21.84 pmol/L) (reference range, 2.0-4.9 pg/mL [SI: 3.07-7.52 pmol/L]) and 3.79 ng/dL (SI: 48.8 pmol/L) (reference range, 0.82-1.63 ng/dL [SI: 10.6-21.0 pmol/L]), respectively, whereas thyroid-stimulating hormone (TSH) was below the detection limit at <0.003 µIU/mL (SI: <0.003 mIU/L) (reference range, 0.61-4.23 µIU/mL [SI: 0.61-4.23 mIU/L]). The TSH receptor antibody level was elevated at 15.7 IU/L (SI: 15.7 IU/L) (reference range, <2.0 IU/L [SI: <2.0 IU/L]), while antinuclear antibodies and antiplatelet antibodies were negative. Ultrasonography revealed diffuse enlargement of the thyroid gland (left lobe: 4.4 SD, right lobe: 5.84 SD) [10] with increased intrathyroidal blood flow (Fig. 1A). Technetium-99m thyroid scintigraphy demonstrated increased thyroid uptake (11.9%) despite a suppressed TSH level, confirming the diagnosis of GD (Fig. 1B).

**Table 1:** Laboratory findings at diagnosis

Laboratory data	Value, conventional unit (SI unit)	Reference range, conventional unit (SI unit)
WBC	1.7 × 10^3^/µL (1.7 × 10^9^/L)	4.5 × 10^3^/µL (4.5 × 10^9^/L) to 13.0 × 10^3^/µL (13.0 × 10^9^/L)
Neutrophil	0.36 × 10^3^/µL (0.36 × 10^9^/L)	1.5 × 10^3^/µL (1.5 × 10^9^/L) to 8.0 × 10^3^/µL (8.0 × 10^9^/L)
Lymphocyte	0.98 × 10^3^/µL (0.98 × 10^9^/L)	1.1 × 10^3^/µL (1.1 × 10^9^/L) to 4.5 × 10^3^/µL (4.5 × 10^9^/L)
Monocyte	0.36 × 10^3^/µL (0.36 × 10^9^/L)	0.2 × 10^3^/µL (0.2 × 10^9^/L) to 1.0 × 10^3^/µL (1.0 × 10^9^/L)
RBC	4.65 × 10^6^/µL (4.65 × 10^12^/L)	3.8 × 10^6^/µL (3.8 × 10^12^/L) to 4.8 × 10^6^/µL (4.8 × 10^12^/L)
Hb	12.9 g/dL (129 g/L)	12.0 g/dL (120 g/L) to 16.0 g/dL (160 g/L)
Platelets	8.0 × 10^4^/µL (80.0 × 10^9^/L)	13.0 × 10^4^/µL (130 × 10^9^/L) to 40.0 × 10^4^/µL (400 × 10^9^/L)
CRP	0.06 mg/dL (0.6 mg/L)	<0.3 mg/dL (<3.0 mg/L)
Alb	4.2 g/dL (42 g/L)	3.9 g/dL (39 g/L) to 4.9 g/dL (49 g/L)
AST	28 U/L (28 U/L)	8 U/L (8 U/L) to 38 U/L (38 U/L)
ALT	24 U/L (24 U/L)	4 U/L (4 U/L) to 43 U/L (43 U/L)
LDH	188 U/L (188 U/L)	124 U/L (124 U/L) to 222 U/L (222 U/L)
CK	44 U/L (44 U/L)	32 U/L (32 U/L) to 187 U/L (187 U/L)
BUN	10.3 mg/dL (3.7 mmol/L)	8.5 mg/dL (3.0 mmol/L) to 20 mg/dL (7.1 mmol/L)
Cre	0.32 mg/dL (28 µmol/L)	0.36 mg/dL (32 µmol/L) to 1.06 mg/dL (94 µmol/L)
Na	140 mmol/L (140 mmol/L)	135 mmol/L (135 mmol/L) to 147 mmol/L (147 mmol/L)
K	3.7 mmol/L (3.7 mmol/L)	3.6 mmol/L (3.6 mmol/L) to 5.5 mmol/L (5.5 mmol/L)
Cl	103 mmol/L (103 mmol/L)	98 mmol/L (98 mmol/L) to 108 mmol/L (108 mmol/L)
T-Chol	123 mg/dL (3.2 mmol/L)	120 mg/dL (3.1 mmol/L) to 220 mg/dL (5.7 mmol/L)
FT3	14.22 pg/mL (21.84 pmol/L)	2.0 pg/mL (3.07 pmol/L) to 4.9 pg/mL (7.52 pmol/L)
FT4	3.79 ng/dL (48.8 pmol/L)	0.82 ng/dL (10.6 pmol/L) to 1.63 ng/dL (21.0 pmol/L)
TSH	<0.003 µIU/mL (<0.003 mIU/L)	0.61 µIU/mL (0.61 mIU/L) to 4.23 µIU/mL (4.23 mIU/L)
Thyroglobulin	6.07 ng/mL (6.07 µg/L)	<33.7 ng/mL (<33.7 µg/L)
TRAb	15.7 IU/L (15.7 IU/L)	<2 IU/L (<2 IU/L)
Antinuclear antibody	Negative	
Antiplatelet antibody	Negative	

Abbreviations: Alb, albumin; ALT, alanine aminotransferase; AST, aspartate aminotransferase; BUN, blood urea nitrogen; CK, creatine kinase; Cl, chloride; Cre, creatinine; CRP, C-reactive protein; FT3, free triiodothyronine; FT4, free thyroxine; Hb, hemoglobin; K, potassium; LDH, lactate dehydrogenase; Na, sodium; RBC, red blood cell count; T-Chol, total cholesterol; TRAb, thyrotropin receptor antibody; TSH, thyroid-stimulating hormone; WBC, white blood cell count.

## Treatment

Oral bisoprolol, a selective β1-blocker, was initiated for symptomatic management, after which the neutrophil and platelet counts increased slightly, from 0.36 × 10^3^/µL (SI: 0.36 × 10^9^/L) to 0.66 × 10^3^/µL (SI: 0.66 × 10^9^/L) and from 8.0 × 10^4^/µL (SI: 80.0 × 10^9^/L) to 9.8 × 10^4^/µL (SI: 98.0 × 10^9^/L), respectively. Potassium iodide (KI) therapy was subsequently initiated, after confirming the diagnosis by technetium-99m thyroid scintigraphy on day 5, resulting in a decrease in FT4 levels, and this was associated with further recovery of neutrophil and platelet counts. On day 8, after confirming recovery of the neutrophil count to >1.0 × 10^3^/µL (SI: 1.0 × 10^9^/L), methimazole (MMI) was initiated at 7.5 mg/day. The platelet count increased to 18.5 × 10^4^/µL (SI: 185 × 10^9^/L) on day 11, and the petechiae gradually resolved. Bisoprolol and KI were sequentially discontinued at day 14 and day 25, respectively. Blood cell counts remained within the normal range thereafter, and thyroid function has remained well controlled with MMI monotherapy. The clinical course is shown in Fig. 1C.

## Outcome and follow-up

At 6 months after discharge, the neutrophil count was 1.8 × 10^3^/µL (SI: 1.8 × 10^9^/L), platelet count was 19.8 × 10^4^/µL (SI: 198 × 10^9^/L), and FT4 was 1.01 ng/dL (SI: 13.0 pmol/L) on MMI 7.5 mg/day, with no recurrence of cytopenia.

## Discussion

We herein report a 12-year-old girl with GD who presented with agranulocytosis and thrombocytopenia at diagnosis. Antinuclear and antiplatelet antibodies were both negative, and cytopenias recovered in parallel with the improvement of thyrotoxicosis. These results suggest that the cytopenias were unlikely to be due to concomitant autoimmune mechanisms and that there is a possible association between thyrotoxicosis and cytopenias.

The frequency of neutropenia at the time of diagnosis of GD has been reported to be approximately 10% to 14.1% in adults [4, 7]. In contrast, data in pediatric patients are limited. Schempp et al [8] reported that 9 of 161 untreated pediatric patients with GD (5.6%) presented with neutropenia at diagnosis, defined as a neutrophil count of <1.8 × 10^3^/µL (SI: 1.8 × 10^9^/L). When limited to cases complicated by agranulocytosis (neutrophil count <0.5 × 10^3^/µL [SI: 0.5 × 10^9^/L]) at diagnosis, only a single pediatric case has been reported [2]. The reported case with agranulocytosis is summarized in Table 2. Similar to the present case, neutrophil counts improved after the initiation of a β-blocker, after which MMI was safely introduced.

**Table 2:** Summary of previous publications reporting agranulocytosis and/or thrombocytopenia

Author, year (ref. no.)	Age, sex	Thyroid function test, conventional unit (SI unit)			Hematology, conventional unit (SI unit)			ITP	Treatment
		TSH	FT4	FT3	Neutrophil	Platelets	Hemoglobin		
Lee et al, 2003 (9)	10, F	<0.001 µIU/mL (<0.001 mIU/L)	>7.8 ng/dL (>100 pmol/L)	NA	Normal	2.8 × 10^4^/µL (28 × 10^9^/L)	12.9 g/dL (129 g/L)	No	Thrombocytopenia improved by carbimazole
Litao et al, 2021 (2)	4, F	NA	1.79 ng/dL (23.0 pmol/L)	6.1 pg/mL (9.37 pmol/L)	0.40 × 10^3^/µL (0.40 × 10^9^/L)	NA	NA	No	Neutropenia improved with β-blocker, followed by successful treatment with MMI
Faienza et al, 2020 (11)	14, F	<0.01 µIU/mL (<0.01 mIU/L)	6.27 ng/dL (80.7 pmol/L)	NA	Normal	3.4 × 10^4^/µL (34 × 10^9^/L)	Normal	Yes	Thrombocytopenia improved by MMI and IVIG
Present case	12, F	<0.003 µIU/mL (<0.003 mIU/L)	3.79 ng/dL (48.8 pmol/L)	14.22 pg/mL (21.84 pmol/L)	0.36 × 10^3^/µL (0.36 × 10^9^/L)	8.0 × 10^4^/µL (80 × 10^9^/L)	12.9 g/dL (129 g/L)	No	Neutropenia and thrombocytopenia improved with β-blocker and KI, followed by successful treatment with MMI

Reference ranges were as follows: TSH, 0.61 to 4.23 µIU/mL (0.61-4.23 mIU/L); FT4, 0.82 to 1.63 ng/dL (10.6-21.0 pmol/L); FT3, 2.0 to 4.9 pg/mL (3.07-7.52 pmol/L); neutrophils, 1.5 to 8.0 × 10^3^/µL (1.5-8.0 × 10^9^/L); platelets, 13.0 to 40.0 × 10^4^/µL (130-400 × 10^9^/L); and hemoglobin, 12.0 to 16.0 g/dL (120-160 g/L).

Abbreviations: FT3, free triiodothyronine; FT4, free thyroxine; ITP, immune thrombocytopenia; IVIG, intravenous immunoglobulin; MMI, methimazole; NA, not available; TSH, thyroid-stimulating hormone.

Thrombocytopenia at the time of diagnosis has been reported to be found in 43% of adults with untreated hyperthyroidism [5]. In the pediatric population, however, no comprehensive studies have been published, and only 3 cases, including the present case, have been described in the literature [9, 11]. These cases are summarized in Table 2. All reported patients exhibited bleeding manifestations, and 1 case was complicated by immune thrombocytopenic purpura and intravenous immunoglobulin therapy was administered. In the remaining subjects, antithyroid drugs were selected as the primary treatment with successful recovery of platelet counts.

The mechanisms of neutropenia in GD remain to be elucidated. Immune-mediated mechanisms have been proposed. For example, Weitzman et al [3] reported that TSH receptor antibodies bind to TSH receptors expressed on neutrophils, leading to opsonization and complement activation. However, accumulating clinical observations suggest that thyrotoxicosis per se may also play an important role in the development of neutropenia. In the current case, neutropenia recovered promptly after treatment of thyrotoxicosis with KI, without the use of MMI. Consistent with our case, Gangadharan et al [12] described a 13-year-old boy with GD who presented with neutropenia (neutrophil count <1.5 × 10^3^/µL [SI: 1.5 × 10^9^/L]) prior to treatment, which was successfully managed with Lugol iodine and a β-blocker. In addition, there is a report showing that neutropenia can recover even without antithyroid drug therapy [13].

Mechanistically, several lines of evidence support the direct impact of excess thyroid hormone on hematopoiesis. Excess thyroid hormone has been shown to affect the proliferation of hematopoietic progenitor cells [14, 15]. Kawa et al [14] reported that hyperthyroidism induced apoptosis in human CD34(+)-enriched hematopoietic progenitor cells obtained from peripheral blood. Taken together, these findings suggest that thyrotoxicosis itself can contribute to GD-related neutropenia, although immune-mediated mechanisms may also operate in some patients.

There might be an additional level of regulation of neutropenia. There is evidence that serum E-selectin levels are higher in individuals with GD complicated by leukopenia than in those without leukopenia, and that leukocyte count is negatively correlated with E-selectin levels. Although a causal relationship was not evident in this study, the authors speculated that neutrophil adhesion to the vascular endothelium is accelerated in GD, leading to an apparent reduction in circulating neutrophil counts [16].

The mechanistic basis of thrombocytopenia in GD also remains to be elucidated. Immune thrombocytopenia has been reported in association with autoimmune thyroid disease, suggesting an autoimmune-mediated mechanism. However, several reports have described simultaneous recovery of platelet counts with improvement of thyrotoxicosis after antithyroid drug therapy [9, 17, 18]. Although antithyroid drugs may have immunosuppressive effects, immunomodulation typically requires a longer time course. Therefore, the rapid, parallel improvement in platelet counts suggests that thrombocytopenia may be related to the severity of thyrotoxicosis itself. Our case supports this interpretation because the platelet count recovered after KI treatment without antithyroid drug therapy. These observations indicate that, at least in some patients, thrombocytopenia in GD may be driven primarily by thyrotoxicosis rather than by an autoimmune mechanism alone. Indeed, there is emerging evidence to support this notion. Xu et al [19] have recently shown that an excess thyroid hormone exposure in human cord blood CD34+ cell-derived megakaryocytes induced megakaryocyte apoptosis via autophagy-related pathways, leading to decreased platelet production.

In the present case, cytopenia partially improved after the initiation of a β-blocker. These findings may suggest that activation of the sympathetic nervous system is involved in the pathogenesis of cytopenia associated with GD. Stimulation by the sympathetic nervous system is considered to regulate the distribution and trafficking of blood cells within the body and may thereby alter peripheral blood cell counts. Ince et al [20] showed that sympathetic input to the bone marrow can regulate the retention factor (C-X-C motif chemokine ligand 12 [CXCL12]) via receptors and may control the release of hematopoietic stem/progenitor cells and leukocytes from the bone marrow. Furthermore, Katayama et al [21] experimentally demonstrated that sympathetic stimulation regulates the mobilization of hematopoietic stem/progenitor cells through downregulation of the retention factor in mice. From these findings, sympathetic activation associated with GD may contribute to cytopenia, including neutropenia, by inducing changes in the bone marrow microenvironment and leukocyte dynamics.

An important clinical question in this case is whether antithyroid drug therapy should be deferred in patients with GD presenting with severe pretreatment cytopenia. Pretreatment neutropenia in GD does not necessarily predict subsequent MMI-induced agranulocytosis and is often attributable to thyrotoxicosis itself; therefore, it may improve after treatment of hyperthyroidism with antithyroid drugs. Indeed, the 2016 American Thyroid Association guidelines note that there is no evidence that preexisting neutropenia increases the risk of complications from antithyroid drugs [22]. However, the same guidelines also state that a baseline absolute neutrophil count <1.0 × 10^3^/µL (SI: 1.0 × 10^9^/L) should prompt serious reconsideration of initiating antithyroid drug therapy [22]. In the present case, the patient had severe neutropenia, with an absolute neutrophil count of 0.36 × 10^3^/µL (SI: 0.36 × 10^9^/L), accompanied by thrombocytopenia and fever. We therefore selected KI as a short-term bridging therapy to control thyrotoxicosis while awaiting hematologic recovery. Importantly, KI was not intended as definitive or prolonged therapy, because prolonged iodine administration may result in escape from the Wolff–Chaikoff effect [22]. Accordingly, MMI was introduced promptly after neutrophil recovery, and KI was subsequently discontinued.

In conclusion, we report a pediatric case of GD complicated by severe neutropenia and thrombocytopenia that was initially managed with a β-blocker and KI, followed by MMI after hematologic recovery. This case highlights that, although antithyroid drugs remain the first-line treatment for pediatric GD [22-24], individualized management may be warranted in patients presenting with severe pretreatment cytopenia. In selected patients, KI may serve as a temporary bridging therapy to control thyrotoxicosis until antithyroid drug therapy can be initiated.

## Learning points

- Graves disease complicated by agranulocytosis and thrombocytopenia at diagnosis is rare; however, these hematological abnormalities may be attributable to the disease itself.
- In pediatric GD with cytopenia, initial treatment with a β-blocker and KI may facilitate recovery of blood cell counts.
- Monitoring blood cell counts at the time of diagnosis is important in patients with GD.

## Data Availability

Data sharing is not applicable to this article as no datasets were generated or analyzed during the current study.
